# Co-Design and Mixed-Methods Evaluation of a Digital Diabetes Education Intervention for Nursing Homes: Study Protocol

**DOI:** 10.3390/nursrep15060188

**Published:** 2025-05-27

**Authors:** Stephanie Craig, Tara Anderson, Patrick Stark, Christine Brown Wilson, Gillian Carter, Claire T. McEvoy, Laura Creighton, Elizabeth Henderson, Shannon Porter, Fadwa Alhalaiqa, Erin P. Ferranti, Komal Patel Murali, Yaguang Zheng, Roberta Sammut, Marwa Mamdouh Shaban, Hon-Lon Tam, Norbert Buzás, Don M. Leidl, Gary Mitchell

**Affiliations:** 1 School of Nursing and Midwifery, Queen’s University Belfast, Belfast BT9 7BL, UK; s.craig@qub.ac.uk (S.C.); tanderson@qub.ac.uk (T.A.); p.stark@qub.ac.uk (P.S.); c.brownwilson@qub.ac.uk (C.B.W.); g.carter@qub.ac.uk (G.C.); laura.creighton@qub.ac.uk (L.C.); elizabeth.henderson@qub.ac.uk (E.H.); s.porter@qub.ac.uk (S.P.); 2 Centre for Public Health, Queen’s University Belfast, Belfast BT12 6BA, UK; c.mcevoy@qub.ac.uk; 3 College of Nursing, Qatar University, Doha P.O. Box 2713, Qatar; f.alhalaiqa@qu.edu.qa; 4 Nell Hodgson Woodruff School of Nursing, Emory University, Atlanta, GA 30322, USA; epoe@emory.edu; 5 College of Nursing, New York University, New York, NY 10010, USA; kp47@nyu.edu (K.P.M.); yaguang.zheng@nyu.edu (Y.Z.); 6 Faculty of Health Sciences, University of Malta, MSD2080 Msida, Malta; roberta.sammut@um.edu.mt; 7 Faculty of Nursing, Cairo University, Cario 4240310, Egypt; marwa.mamdouh@cu.edu.eg; 8 The Nethersole School of Nursing, Faculty of Medicine, The Chinese University of Hong Kong, Hong Kong 999077; hltam@cuhk.edu.hk; 9 Faculty of Health Sciences and Social Studies, University of Szeged, H-6726 Szeged, Hungary; buzas.norbert@med.u-szeged.hu; 10 Faculty of Nursing, University of New Brunswick, Moncton, NB E1C 0L2, Canada; don.leidl@unb.ca

**Keywords:** co-design, diabetes, digital, education, health, intervention, nursing homes, older people

## Abstract

**Background:** Diabetes is common among nursing home residents, with approximately one in four affected, a figure expected to rise. Despite the complexity of care required, educational support for nursing home staff remains limited. This study will aim to co-design and evaluate a digital intervention to improve staff knowledge, confidence, and practices in diabetes care. **Methods:** The study will follow a logic model across three workstreams. Workstream 1 (WS1) will inform the model inputs through three phases: (1) a scoping review will be conducted to summarise existing diabetes education initiatives in nursing home settings; (2) approximately 20 semi-structured interviews will be carried out with nursing home staff to explore perceived barriers and supports in delivering diabetes care; and (3) a modified Delphi process involving 50–70 diverse stakeholders will be used to establish educational priorities. Workstream 2 (WS2) will involve co-designing a digital diabetes education intervention, informed by WS1 findings. Co-design participants will include nursing home staff, diabetes professionals, and people living with diabetes or their carers. Workstream 3 (WS3) will consist of a mixed-methods evaluation of the intervention. Pre- and post-intervention questionnaires will assess staff knowledge, confidence, and attitudes. The usability of the intervention will also be measured. Following implementation, focus groups with approximately 32 staff members will be conducted to explore user experiences and perceived impact on resident care. **Discussion:** This study will address an important gap in staff education and support, aiming to improve diabetes care within nursing home settings through a digitally delivered, co-designed intervention.

## 1. Introduction

Diabetes is a chronic and increasingly prevalent global health issue, affecting approximately 537 million people (10.5% of the global population), with projections rising to 643 million by 2030 and 783 million by 2045, predominantly in low- and middle-income countries (LMICs) where healthcare systems often struggle to manage the burden [1,2]. It is characterised by elevated blood glucose levels due to insufficient insulin production or ineffective insulin use [3,4], and includes Type 1, Type 2, gestational, and rarer forms like monogenic diabetes and latent autoimmune diabetes in adults (LADA) [5]. Type 1 diabetes is autoimmune, while Type 2, accounting for 90–95% of cases, is linked to obesity, inactivity, and unhealthy diets [6,7], and though manageable with lifestyle changes and medication, often leads to serious complications such as cardiovascular disease and kidney problems [3]. The condition imposes a massive economic burden, with diabetes-related healthcare costs reaching USD 966 billion and expected to exceed USD 1 trillion by 2045 [2]; globally, the economic impact exceeds USD 760 billion [8], and individuals with diabetes incur healthcare costs 2.3 times higher than those without the condition [9]. Without effective intervention, the global diabetes burden will continue to grow, demanding coordinated international action to improve healthcare access, encourage healthier lifestyles, and implement preventive strategies [4].

The rise in life expectancy and the growing number of older adults has contributed to the increasing prevalence of diabetes in nursing homes, where up to 25–34% of residents have the condition [10]. Older adults with diabetes are often managing multiple comorbidities, such as cardiovascular disease, neuropathy, nephropathy, and cognitive decline, making diabetes management more complex [11]. In addition, many nursing home residents have diminished functional capacity, frailty, and varying degrees of cognitive impairment, which exacerbate the challenges of diabetes care [12]. This complexity highlights the need for individualised care plans that address not only the medical aspects of diabetes but also the unique needs of each resident, including mobility, nutrition, and cognitive health [13], while recognising the essential role of non-pharmacological interventions in complementing effective medications to support holistic care.

Diabetes management in nursing homes involves regular blood glucose monitoring, appropriate medication administration, and tailored nutritional interventions. These strategies are essential not only for preventing acute complications, such as hypoglycaemia and hyperglycaemia, but also for mitigating long-term risks, such as cardiovascular disease and neuropathy [4,14]. Nursing home staff play a critical role in managing diabetes for their residents, yet research has shown that many staff members lack the necessary education and training to effectively manage the condition [15]. This knowledge gap can lead to suboptimal care and poorer outcomes for residents living with diabetes [16]. Furthermore, the increasing prevalence of diabetes in nursing homes has highlighted the importance of preventive interventions aimed at reducing the onset of diabetes. Lifestyle modifications, such as diet and exercise, have been shown to play a crucial role in maintaining healthy glucose levels and preventing the progression from prediabetes to Type 2 diabetes [17]. For nursing home residents, facilitating regular physical activity and providing nutritious meals tailored to individual dietary needs can significantly improve health outcomes and overall well-being [16].

Despite the known challenges and complexities of diabetes management in nursing homes, there remains a critical need for enhanced diabetes education and training for nursing home staff. Comprehensive education on diabetes can equip staff with the necessary knowledge to administer medications correctly, monitor glucose levels effectively, and respond to changes in residents’ health status [18,19]. Additionally, empowering residents to take part in their own diabetes management, where feasible, can promote independence and improve quality of life [20]. Given the growing number of nursing home residents living with diabetes and the associated healthcare challenges, there is an urgent need to develop and implement interventions that support effective diabetes management in these settings [17,21]. In the United Kingdom, nursing homes provide long-term care for older adults with complex health needs, including those with diabetes [22]. Admission to a nursing home typically follows a comprehensive assessment of an individual’s medical, functional, and social needs, often initiated by healthcare professionals or social services in consultation with families [23]. Nursing homes are staffed by a multidisciplinary team, including registered nurses, care assistants, managers, and, in some settings, allied health professionals such as occupational therapists and physiotherapists [23]. Staff educational backgrounds vary, with registered nurses holding professional qualifications and care assistants typically having vocational training [24].

A key area of focus is the co-design and testing of digital interventions, which can provide real-time support and education for both nursing home staff and residents. Digital tools hold significant potential to improve diabetes management by facilitating continuous monitoring, delivering tailored educational content, and supporting individualised care plans [25]. By addressing the unique challenges of managing diabetes in nursing homes, these interventions may help optimise the quality of life for residents and improve overall care outcomes. Given the non-clinical nature of this intervention, its effects will be evaluated using validated self-report instruments measuring changes in self-efficacy, knowledge, and communication practices. This approach allows for understanding of behavioural and attitudinal shifts that may result from the educational content. There is a notable paucity of educational interventions specifically designed to support care home staff in managing diabetes, with digital approaches being even more scarce. This study addresses this gap by developing and evaluating a novel, co-designed digital intervention tailored to the unique needs of nursing home settings.

Therefore, the aim of this study is to co-design and evaluate a digital intervention to improve nursing home staff knowledge, self-efficacy (confidence, as measured by a validated self-efficacy questionnaire), and practice related to the care of residents living with diabetes.

## 2. Methods

### 2.1. Design

Logic modelling will guide the design of this interventional study, facilitating improved research planning by identifying theoretical and practical gaps and informing decisions regarding data collection and analysis methods [26]. This study will follow best practices as outlined in the NIHR framework for complex interventions [27], unfolding across three primary workstreams: theorisation, where we will integrate established theories to inform our intervention’s design; co-design of the digital intervention, which will involve engaging patients and experts to collaboratively develop the intervention based on insights from the theorisation phase; and mixed-methods evaluation, focusing on proximal outcome testing to assess the intervention’s effectiveness. By embedding theory into each stage of the process, we aim to ensure that our intervention is not only tailored to meet identified needs but also rigorously evaluated [25], ultimately contributing to a robust evidence base for effective health interventions. As this study is not a clinical trial, it has not been formally registered.

**Workstream 1: Theorisation**

Workstream 1 (WS1) will contribute to the ‘inputs’ of the logic model and will be executed in three phases. The first two phases will occur concurrently, followed by the third phase upon their completion. WS1 is expected to be completed within the first 12 months of the study. Its aim is to gather evidence at global (scoping review), national (Delphi study), and local (qualitative interviews) levels to inform the development of the digital educational intervention.

**WS1 phase 1: Scoping review**

Phase 1 will involve conducting a scoping review of the literature to synthesise existing evidence on interventions within nursing homes aimed at enhancing care for residents with diabetes. A review protocol can be found here: https://osf.io/ge7nq/ (accessed on 24 April 2025). This phase will address the following objectives:

- What interventions have been implemented, if any, to improve diabetes care for residents in nursing homes?
- Have these interventions proven effective in improving diabetes care in nursing homes?
- What are the reported experiences of nursing home staff in delivering interventions for improving diabetes care, including the challenges, facilitators, and barriers they have encountered?

The findings from this review will inform both WS2 and WS3. The review will be conducted in accordance with Joanna Briggs Institute [28] recommendations and the PRISMA-ScR reporting standards for scoping reviews [29]. Searches will be conducted in the following databases: CINAHL Plus, Medline, PubMed and PsycINFO. Example search terms will include ‘diabetes’, ‘diabetic’, ‘nursing homes’, ‘residential homes’, ‘education’, and ‘training.’ Reference lists of relevant systematic and scoping reviews will be manually searched to identify additional studies not located in the initial search. The review will include studies that evaluate diabetes education interventions for nursing home staff, regardless of delivery mode, provided they report outcomes such as improvements in staff knowledge or resident quality of life. Studies focusing solely on outcomes related to diabetes management (e.g., drug prescribing and symptom monitoring) will be excluded, as will non-interventional studies, studies lacking a clear association with diabetes, and studies not conducted exclusively in nursing home settings.

**WS1 phase 2: Qualitative Interviews with Nursing home Staff**

Individual, semi-structured qualitative interviews will be arranged with staff members working in both nursing and residential care homes. The primary aim of these interviews is to explore the factors that support or hinder the delivery of care to residents living with diabetes. All interviews will be conducted remotely using Microsoft Teams to facilitate participation and accessibility. Qualitative data gathered will provide an understanding of current challenges and enablers, directly informing the collaborative design process for the digital intervention in Workstream 2 (WS2).

Consistent with best practice guidance for qualitative research, we plan to recruit around 20 participants. Recruitment will continue until thematic saturation is achieved, ensuring a comprehensive capture of relevant perspectives. Staff will be invited to participate via convenience sampling from the Northern Ireland Nursing Home Research Network at Queen’s University Belfast (QUB), which comprises over 400 registered nurses active in the sector. This network’s members have previously agreed to be contacted for research, quality improvement, or educational activities. Potential participants will be approached through email invitations. Nursing home staff are defined as all employees providing direct care to residents, including registered nurses and care assistants who are involved in direct resident care.

The participants’ levels of experience are expected to vary, with eligible participants including home managers, charge nurses, registered nurses, care assistants who provide direct care to residents with diabetes. To be eligible, participants must have been practising within the nursing home setting for more than six months and provide nursing care to residents living with diabetes.

**WS1 phase 3: Delphi study**

A modified Delphi study will be conducted to identify the key educational priorities related to diabetes, as perceived by relevant stakeholders. This study will build on findings from the preceding scoping review and individual interviews. The initial Delphi items will be developed by the research team based on empirical data obtained during phases 1 and 2 of Workstream 1 (WS1). The Delphi method is a structured process for eliciting expert opinion through multiple rounds of questionnaires, allowing for anonymous responses and iterative reflection. This approach facilitates the development of consensus on complex issues through controlled feedback [30].

The study will consist of three rounds of online surveys administered to stakeholders across the United Kingdom (England, Scotland, Wales, and Northern Ireland). Stakeholder groups will include nursing home staff, diabetes practitioners, individuals living with diabetes, carers, educationalists, community dietitians working in care home settings, and representatives from relevant charitable organisations. Recruitment will be supported by the Royal College of Nursing’s Older People Forum, a UK-wide professional network comprising approximately 12,000 members involved in the care of older adults. Additional participants will be recruited via the research team’s professional networks.

Based on prior Delphi studies, it is expected that a sample of 50 to 70 stakeholders will be recruited [25]. Surveys will be administered using Microsoft Forms and distributed to participants who have provided informed consent. Following the completion of the third survey round, an online consensus meeting will be held. During this meeting, stakeholders will anonymously vote on the educational priorities to be used in the co-design of the digital intervention developed in Workstream 2 (WS2). The Delphi process is expected to be completed over a six-month period, allowing time for survey distribution, response collection, and data analysis.

**Workstream 2: Co-design of the digital intervention**

Guided by the findings from Workstream 1 (WS1), Workstream 2 (WS2) will centre on the co-design and development of a digital intervention. The co-design methodology entails iterative and collaborative engagement between the research team, software developers, and end-users [31,32,33,34,35], ensuring alignment between technological functionality and user requirements. This collaborative approach is intended to enhance the usability, acceptability, and overall effectiveness of the intervention [36]. The digital intervention will constitute the primary ‘output’ within the corresponding logic model framework.

The co-design process will involve approximately 20 participants, comprising a balanced representation of key stakeholder groups: five individuals with lived experience of diabetes or their carers, five healthcare professionals with diabetes expertise, five staff members from nursing homes, and five members of the research team with clinical or academic expertise in diabetes care and residential aged care settings. Participant recruitment will be managed by the research team to ensure broad stakeholder representation. An external technology partner will be actively engaged throughout the co-design process, attending all sessions and offering real-time input on prototype development and refinement.

The co-design group will convene once per month over a four-month period. The initial session will introduce participants, facilitate knowledge-sharing based on lived and professional experiences, and present key insights from WS1. The second session will prioritise identifying core objectives and functional requirements of the intervention. The third session will focus on refining content, including ensuring that language is accessible and appropriate for the intended end-user audience. The final session will concentrate on visual and usability aspects, such as layout, navigation structure, and colour palette. These iterative workshops are designed to ensure the intervention is user-centred and contextually appropriate. All meetings are expected to occur in person, in alignment with international best practice guidelines for co-design processes [31,32,33,37,38,39,40,41].

**Workstream 3: Intervention testing**

Workstream 3 (WS3) will involve intervention testing of the digital intervention, using a mixed-methods approach to assess its outcomes, which will form the final phase of the study. The digital intervention will be implemented over a period of 12 weeks, during which nursing home staff will have access to the educational materials and complete pre- and post-intervention assessments.

**WS3 phase 1: Nursing home staff knowledge, self-efficacy, and usability**

In phase 1, nursing home staff knowledge and self-efficacy in providing care for residents with diabetes will be assessed before and after implementing the digital intervention via a pre- and post-test questionnaire. The questionnaire, delivered electronically through the intervention, will include the Michigan Diabetes Knowledge Test (DKT2) (twenty-three items) [42] and an eight-item questionnaire on self-efficacy in providing care to residents with diabetes. The Michigan Diabetes Knowledge Test (DKT2) is a widely validated tool used to assess general diabetes knowledge and has demonstrated good internal consistency (Cronbach’s alpha = 0.77) [42]. The self-efficacy questionnaire was adapted from established scales used in similar care settings, with items reviewed by experts during the co-design process to ensure face validity. Approximately 100 registered nurses will be recruited from ten nursing homes across Northern Ireland. These homes will represent both rural and urban settings. A power calculation will not be required as we plan to conduct proximal testing as per NIHR guidelines on complex interventions [27]. However, the anticipated sample of approximately 100 registered nurses from 10 nursing homes is consistent with recommendations for feasibility and proximal outcome testing in complex intervention research [27]. This sample size allows for preliminary evaluation of changes in knowledge and self-efficacy, while also accounting for expected attrition in this population. Analytics, such as user engagement and time spent on the platform, will also be analysed to evaluate the intervention’s usability. Usability will be further evaluated using the 31-item User Engagement Scale (UES) questionnaire [43], which will address the ‘practicality’ aspect of the testing. The User Engagement Scale (UES) has robust psychometric properties, including strong reliability and construct validity in digital health education contexts [43]. The selection of these tools was guided by their proven validity, relevance to the target population, and alignment with the intervention’s educational objectives.

**WS3 phase 2: Staff experience on implementation**

Phase 2 will focus on understanding nursing home staff experiences of implementing the intervention and their perceived impact of the educational intervention on their practice and resident care. Focus groups (*n* = 4) will be conducted with up to 32 nursing home staff, including managers and nurses. Depending on data saturation, participant numbers may be adjusted. Recruitment will follow the completion of the post-test questionnaires in phase 1, with participants offered the opportunity to join the focus groups.

Table 1 provides an overview of workstreams and phases for this study.

### 2.2. Data Analysis

#### 2.2.1. Qualitative Analysis

Qualitative data will be collected through one-on-one interviews (Workstream 1; WS1) and focus groups (Workstream 3; WS3), both conducted online via Microsoft Teams to enhance participant accessibility and scheduling flexibility. All sessions will be audio recorded in full. The recordings from WS1 and WS3 will be transcribed verbatim and imported into NVivo version 11 for data management and analysis. A thematic analysis approach, as described by Braun and Clarke [44], will be employed to systematically identify, analyse, and report patterns (themes) within the data. One researcher will conduct all individual interviews, while two researchers will co-facilitate each focus group. All researchers involved in the qualitative data collection will have prior training and experience in the respective qualitative methodologies employed.

#### 2.2.2. Quantitative Analysis

Quantitative data will be analysed using IBM SPSS Statistics, version 26. Descriptive statistics will be used to summarise findings from the modified Delphi study (WS1) as well as platform-generated internal analytics collected during the evaluation phase (WS3). These analytics include metrics such as the duration of time care staff engage with the digital intervention. To evaluate changes in nursing home staff knowledge and self-efficacy following exposure to the intervention, dependent (paired samples) *t*-tests will be conducted as part of WS3. This analysis will provide a preliminary indication of the intervention’s potential effectiveness.

#### 2.2.3. Framework

The Consolidated Framework for Implementation Research (CFIR) will be used to systematically guide the evaluation of the digital diabetes education intervention within nursing homes. Specifically, CFIR’s five domains, (1) intervention characteristics, (2) outer setting, (3) inner setting, (4) characteristics of individuals, and (5) implementation process, will inform the development of data collection tools and the analysis of both qualitative and quantitative findings in Workstream 3 [45,46]. For example, interview and focus group questions will be mapped to CFIR constructs to explore how factors such as organisational culture, staff readiness, and external policy influence the adoption and integration of the intervention. This approach will ensure that the evaluation captures key contextual and process-related determinants of successful implementation, enabling identification of barriers and facilitators unique to the nursing home environment [45,46]. Data generated is likely to inform recommendations for scaling and sustaining digital education interventions in similar care settings.

#### 2.2.4. Ethics and Governance

This study has received ethical approval from the Faculty of Medicine and Health Sciences at Queen’s University Belfast (MHLS 24_167). Local governance will be managed by each nursing home or nursing home group, as these organisations operate independently of the National Health Service (NHS) in Northern Ireland. Microsoft Teams, which complies fully with the Data Protection Act [47], will be used for data management and has the capability to record meetings, ensuring thorough capture of all participants’ views. Video recordings will be securely stored and deleted following transcription. Informed consent will be collected from all participants, and their involvement will be kept confidential. Participant information will be accessible only to the research team, and participants’ identities will be anonymised in all publications and study outputs. The study will be conducted in accordance with the Declaration of Helsinki [48]. All data will be stored on password-protected computers at Queen’s University Belfast and anonymised transcription files on a secure Microsoft Teams site accessible only to the research team. All data will comply with the Data Protection Act [47] and be retained for 5 years post-study before being destroyed.

## 3. Discussion

There is a significant gap in research focused on the development and testing of digital interventions aimed at improving care staff understanding of diabetes management and symptom monitoring in nursing homes. As the prevalence of diabetes continues to rise, particularly among older adults, the need for effective training and support for care staff has never been more critical [49]. A systematic review of 63 interventional studies designed to change care staff practices in nursing homes revealed that while achieving change is feasible, the process is inherently complex [50]. This complexity is influenced by various factors, including staff attitudes, existing knowledge levels, and the organisational culture within nursing homes. Compared to similar digital educational interventions in long-term care, this study is distinguished by its co-design methodology involving diverse stakeholders including residents, carers, and frontline staff. While other studies have focused on clinical or face-to-face training formats, few have integrated participatory design into digital diabetes education in nursing home settings, highlighting the innovation and originality of this approach. This study aims to actively engage end users through a multi-faceted approach, including one-on-one interviews, a Delphi study, a co-design group, and focus groups. This inclusive methodology not only ensures that the voices of care staff are heard but also fosters a sense of ownership and commitment to the intervention being developed [25,51].

The outcomes of this study are proximal in nature and focus on the extent to which the intervention impacts knowledge, self-efficacy, acceptability, care practice, and short-term resident outcomes. While initial findings may be promising, expectations should remain cautious and grounded in the early-stage nature of this work. Larger, randomised studies will be required to validate these effects and determine long-term impacts. Should these outcomes be positive, further testing could test medium- and longer-term outcomes which may lead to sustained improvements. Furthermore, if the intervention proves successful, it holds the potential for scalability at both national and international levels. This scalability could be achieved through partnerships with healthcare organisations and policymakers who recognise the importance of quality diabetes care in long-term care settings. Given the high staff turnover in healthcare [52,53,54], adopting a digital approach for training presents a more affordable and sustainable solution compared to traditional face-to-face methods [55]. Digital platforms offer flexibility [41,56], allowing training to be standardised and readily accessible to new staff as they onboard.

There are methodological limitations within this study protocol that should also be acknowledged. The study does not use randomisation, which may introduce selection bias. Furthermore, the follow-up period is relatively short and may not capture sustained behavioural changes or long-term impacts on resident outcomes. The participant sample may also lack diversity in terms of geographical and cultural representation. These limitations should be considered when interpreting the findings and their generalizability.

It is anticipated that the intervention developed during this study will not only impact care provision for residents with diabetes in nursing homes but, if successful, could also have scalability at both a national and international level in the future. However, practical challenges such as the need for staff training in digital literacy, inconsistent access to devices and internet connectivity, and potential resistance to change within organisational cultures must be considered. Addressing these barriers will be key to the intervention’s wider adoption and long-term success.

To facilitate impact at an international level, an International Observer Panel (IOP) has been established with nine experts in diabetes, digital education, and care of older people or in nursing home settings (FA, EF, KPM, YZ, RS, MMS, HLT, BN, and DL). Members of the IOP provided guidance and recommendations in the development of this protocol. The IOP will also monitor project activities throughout and provide expert input into each stage of the study. The IOP will also be able to provide feedback about how the intervention could work or might be adapted in their home countries and support international dissemination activities. It is anticipated that members of the IOP will meet with the research team twice per year using Microsoft Teams. Additional email contact will be held with members of the IOP throughout each stage of the study. Members of the IOP will provide expert feedback and consultation on the project and as such, will be acknowledged as co-authors on all outputs associated with the project.

Following the evaluation of this study, the digital education intervention will be freely available to all. The research team is hopeful that this education will support the development in knowledge, improve nursing home nursing self-efficacy in diabetes and improve patient care.

### Implications

This protocol outlines a study to co-design and evaluates a digital diabetes education intervention for nursing home staff. The findings are expected to inform best practices in staff training, improve diabetes management and outcomes for residents, and provide a model for implementing digital education in similar care settings. The intervention, if effective, has the potential for national and international adoption, contributing to improved quality of care in nursing homes.

## Figures and Tables

**Table 1:** Overview of workstreams and phases.

Workstream	Description
Workstream 1: Theorisation	(1a) Scoping review of diabetes interventions (1b) Qualitative interviews with nursing staff (1c) Modified Delphi study with stakeholders
Workstream 2: Co-Design	Co-design meetings with stakeholders including: - People with diabetes and carers - Diabetes professionals - Nursing home staff and researchers
Workstream 3: Intervention Testing	(3a) Pre/post questionnaire testing: - Diabetes knowledge (DKT2) - Self-efficacy - Usability/User Engagement Scale (3b) Focus groups: - Staff experience with the intervention
Workstream 4: Integration and Analysis	Mixed methods integration, interpretation, and preparation for dissemination

## Data Availability

No data are available as this manuscript presents a study protocol.

## Public Involvement Statement

No public involvement in any aspect of this research.

## Guidelines and Standards Statement

This protocol is designed according to the PRISMA Extension for Scoping Reviews (PRISMA-ScR), Consolidated Criteria for Reporting Qualitative Research (COREQ), Conducting and Reporting Delphi Studies (CREDES) guidelines for observational research, and Transparent Reporting of Evaluations with Nonrandomized Designs (TREND) guidelines.

## Abbreviations

The following abbreviations are used in this manuscript:

WS	Workstream
UK	United Kingdom
LMICs	Low- and middle-income countries
LADA	Latent autoimmune diabetes in adults
QUB	Queen’s University Belfast
DKT2	Diabetes Knowledge Test
UES	User Engagement Scale
IOP	International Observer Panel

## References

[1] Hossain M.J., Al-Mamun M., Islam M.R. (2024). Diabetes mellitus, the fastest growing global public health concern: Early detection should be focused. Health Sci. Rep..

[2] International Diabetes Federation (2021). IDF Diabetes Atlas.

[3] World Health Organization (2023). ‘Diabetes’, World Health Organization. https://www.who.int/news-room/fact-sheets/detail/diabetes.

[4] Zheng Y., Ley S.H., Hu F.B. (2018). Global Epidemiology of Type 2 Diabetes and Its Cardiovascular Implications. Nat. Rev. Cardiol..

[5] (2022). American Diabetes Association Professional Practice Committee. 2. Classification and Diagnosis of Diabetes: Standards of Medical Care in Diabetes—2022. Diabetes Care.

[6] Stoian A.P. (2021). Type 2 Diabetes: From Pathophysiology to Cyber Systems.

[7] Ward-Ongley P. (2024). Fundamentals of diet for type 2 diabetes. J. Community Nurs..

[8] Williams R., Karuranga S., Malanda B., Saeedi P., Basit A., Besançon S., Bommer C., Esteghamati A., Ogurtsova K., Zhang P. (2020). Global and Regional Estimates and Projections of Diabetes-Related Health Expenditure: Results from the International Diabetes Federation Diabetes Atlas. Diabetes Res. Clin. Pract..

[9] Parker E.D., Lin J., Mahoney T., Ume N., Yang G., Gabbay R.A., ElSayed N.A., Bannuru R.R. (2024). Economic Costs of Diabetes in the U.S. in 2022. Diabetes Care.

[10] Idrees T., Castro-Revoredo I.A., Migdal A.L., Moreno E.M., Umpierrez G.E. (2022). Update on the management of diabetes in long-term care facilities. BMJ Open Diabetes Res. Care.

[11] Kirkman M.S., Briscoe V.J., Clark N., Florez H., Haas L.B., Halter J.B., Huang E.S., Korytkowski M.T., Munshi M.N., Odegard P.S. (2012). Diabetes in older adults. Diabetes Care.

[12] Lipska K.J., Ross J.S., Miao Y., Shah N.D., Lee S.J., Steinman M.A. (2015). Potential Overtreatment of Diabetes Mellitus in Older Adults With Tight Glycemic Control. JAMA Intern. Med..

[13] Sugandh F., Chandio M., Raveena F., Kumar L., Karishma F., Khuwaja S., Memon U.A., Bai K., Kashif M., Varrassi G. (2023). Advances in the Management of Diabetes Mellitus: A Focus on Personalized Medicine. Cureus.

[14] National Institute of Diabetes and Digestive and Kidney Diseases (2023). Preventing Type 2 Diabetes. NIDDK. https://www.niddk.nih.gov/health-information/diabetes/overview/preventing-type-2-diabetes.

[15] Walker K.-A., Craig S., Anderson T., Stark P., Brown Wilson C., Carter G., McEvoy C., Creighton L., Henderson E., Porter S. (2025). A scoping review of diabetes educational interventions for nursing homes. BMC Med Educ..

[16] Hurley M.V., Wood J., Smith R., Grant R., Jordan J., Gage H., Anderson L.W., Kennedy B., Jones F. (2020). The feasibility of increasing physical activity in nursing home residents: Active Residents in Nursing homes (ARCH) programme. Physiotherapy.

[17] Sinclair A.J., Bellary S., Dashora U., Abdelhafiz A.H., Rowles S., Reedman L., Turner B., Green M., Forbes A., Middleton A. (2023). Enhancing diabetes care for the most vulnerable in the 21st century: Interim findings of the National Advisory Panel on Nursing home Diabetes (NAPCHD). Diabet Med..

[18] Nazar C.M., Bojerenu M.M., Safdar M., Marwat J. (2015). Effectiveness of diabetes education and awareness of diabetes mellitus in combating diabetes in the United Kigdom; a literature review. J Nephropharmacol..

[19] Ernawati U., Wihastuti T.A., Utami Y.W. (2021). Effectiveness of diabetes self-management education (DSME) in type 2 diabetes mellitus (T2DM) patients: Systematic literature review. J. Public Health Res..

[20] Gómez-Velasco D.V., Almeda-Valdes P., Martagón A.J., Galán-Ramírez G.A., Aguilar-Salinas C.A. (2019). Empowerment of patients with type 2 diabetes: Current perspectives. Diabetes Metab. Syndr. Obes..

[21] Pandya N., Hames E., Sandhu S. (2020). Challenges and Strategies for Managing Diabetes in the Elderly in Long-Term Care Settings. Diabetes Spectr..

[22] Sinclair A., Dunning T., Rodriguez-Mañas L. (2015). Diabetes in older people: New insights and remaining challenges. Lancet Diabetes Endocrinol..

[23] Millar R.J., Diehl C., Blake E., Fakeye O., Kusmaul N. (2024). Nursing home characteristics and resident quality of care outcomes: A scoping review. J. Long-Term Care.

[24] Care Quality Commission Nursing Homes Registration (England) [Internet]. London: GOV.UK. https://www.gov.uk/find-licences/nursing-homes-registration-england.

[25] Tay B.S., Edney S.M., Brinkworth G.D., Cox D.N., Wiggins B., Davis A., Gwilt I., Haveman-Nies A., Ryan J.C. (2021). Co-design of a digital dietary intervention for adults at risk of type 2 diabetes. BMC Public Health.

[26] Funnell S.C., Rogers P.J. (2011). Purposeful Program Theory. Effective use of Theories of Change and Logic Models.

[27] Skivington K., Matthews L., Simpson S.A., Craig P., Baird J., Blazeby J.M., Boyd K.A., Craig N., French D.P., McIntosh E. (2021). A new framework for developing and evaluating complex interventions: Update of Medical Research Council guidance. BMJ.

[28] Joanna Briggs Institute (2017). Checklist for Systematic Reviews and Research Syntheses. https://jbi.global/critical-appraisal-tools.

[29] Tricco A.C., Lillie E., Zarin W., O’Brien K.K., Colquhoun H., Levac D., Moher D., Peters M.D.J., Horsley T., Weeks L. (2018). PRISMA Extension for Scoping Reviews (PRISMA-ScR): Checklist and Explanation. Ann. Intern. Med..

[30] Barrett D., Heale R. (2020). What are delphi studies?. Evid. Based Nurs..

[31] Wilson Brown C., Anderson T., Graham M., Murphy J., Mitchell G., Tuohy D., Barry H.E., Boland P., Birch M., Tierney A. (2024). Co-designing an interprofessional digital education resource on delirium: A student-led approach. BMC Med. Educ..

[32] McLaughlin-Borlace N., Creighton L., Mitchell G. (2024). Championing student participation in co-designing digital education resources: A student experience. Nurse Educ. Today.

[33] Anderson K., Gall A., Butler T., Ngampromwongse K., Hector D., Turnbull S., Lucas K., Nehill C., Boltong A., Keefe D. (2022). Development of key principles and best practices for co-design in health with First Nations Australians. Int. J. Environ. Res. Public Health.

[34] Santin O., McShane T., Hudson P., Prue G. (2019). Using a six-step co-design model to develop and test a peer-led web-based resource (PLWR) to support informal carers of cancer patients. Psychooncology.

[35] Visser F.S., Stappers P.J., van der Lugt R., Sanders E.B.N. (2005). Contextmapping: Experiences from practice. CoDesign.

[36] van Gemert-Pijnen L., Kelders S.M., Kip H., Sanderman R. (2018). Ehealth Research, Theory and Development: A Multidisciplinary Approach.

[37] Slattery P., Saeri A.K., Bragge P. (2020). Research co-design in health: A rapid overview of reviews. Health Res. Policy Syst..

[38] Langley J., Wassall R., Geddis-Regan A., Watson S., Verey A., Mckenna G., Brocklehurst P., Tsakos G. (2022). Putting guidelines into practice: Using co-design to develop a complex intervention based on NG48 to enable care staff to pro-vide daily oral care to older people living in nursing homes. Gerodontology.

[39] O’Cathain A., Croot L., Sworn K., Duncan E., Rousseau N., Turner K., Yardley L., Hoddinott P. (2019). Taxonomy of approaches to developing interventions to improve health: A systematic methods overview. Pilot Feasibility Stud..

[40] Green T., Bonner A., Teleni L., Bradford N., Purtell L., Douglas C., Yates P., MacAndrew M., Dao H.Y., Chan R.J. (2020). Use and reporting of experience-based codesign studies in the healthcare setting: A systematic review. BMJ Qual. Saf..

[41] Messiha K., Chinapaw M.J.M., Ket H.C.F.F., An Q., Anand-Kumar V., Longworth G.R., Chastin S., Altenburg T.M. (2023). Systematic review of contemporary theories used for co-creation, co-design and co-production in public health. J. Public Health.

[42] Fitzgerald J.T., Funnell M.M., Anderson R.M., Nwankwo R., Stansfield R.B., Piatt G.A. (2016). Validation of the Revised Brief Diabetes Knowledge Test (DKT2). Diabetes Educ..

[43] O’brien H.L., Cairns P., Hall M. (2018). A practical approach to measuring user engagement with the refined user engagement scale (UES) and new UES short form. Int. J. Human Computer Stud..

[44] Braun V., Clarke V. (2021). Thematic Analysis: A Practical Guide.

[45] Damschroder L.J., Aron D.C., Keith R.E., Kirsh S.R., Alexander J.A., Lowery J.C. (2009). Fostering implementation of health services research findings into practice: A consolidated framework for advancing implementation science. Implement Sci..

[46] Kirk M.A., Kelley C., Yankey N., Birken S.A., Abadie B., Damschroder L. (2016). A systematic review of the use of the Consolidated Framework for Implementation Research. Implement Sci..

[47] Data Protection Act 2018, c. 12. https://www.legislation.gov.uk/ukpga/2018/12/contents/enacted.

[48] World Medical Association (2001). World Medical Association Declaration of Helsinki. Ethical principles for medical research involving human subjects. Bull. World Health Organ..

[49] Sarre S., Maben J., Aldus C., Schneider J., Wharrad H., Nicholson C., Arthur A. (2018). The challenges of training, support and assessment of healthcare support workers: A qualitative study of experiences in three English acute hospitals. Int. J. Nurs. Stud..

[50] Low L.-F., Fletcher J., Goodenough B., Jeon Y.-H., Etherton-Beer C., MacAndrew M., Beattie E. (2015). A systematic review of interventions to change staff care practices in order to improve resident outcomes in nursing homes. PLoS ONE.

[51] McMahon J., Thompson D.R., Brown Wilson C., Hill L., Tierney P., Cameron J., Yu D.S.F., Moser D.K., Spilsbury K., Srisuk N. (2024). Determining the Key Education Priorities Related to Heart Failure Care in Nursing Homes: A Modified Delphi Approach. Healthcare.

[52] Mitchell G., Cousins C., Burrows R., Cousins G. (2017). A review of safe-staffing models and their applicability to nursing homes. J. Nurs. Manag..

[53] Willard-Grace R., Knox M., Huang B., Hammer H., Kivlahan C., Grumbach K. (2019). Burnout and Health Care Workforce Turnover. Ann. Fam Med..

[54] Gandhi A., Yu H., Grabowski D.C. (2021). High nursing staff turnover in nursing homes offers important quality information: Study examines high turnover of nursing staff at US nursing homes. Health Affairs.

[55] Loureiro F., Sousa L., Antunes V. (2021). Use of Digital Educational Technologies among Nursing Students and Teachers: An Exploratory Study. J. Pers. Med..

[56] Booth R.G., Strudwick G., McBride S., O’Connor S., Solano López A.L. (2021). How the nursing profession should adapt for a digital future. BMJ.

